# A Novel Route to Optimize Placement Equipment Kinematics by Coupling Capacitive Accelerometers

**DOI:** 10.3390/s22093423

**Published:** 2022-04-29

**Authors:** João Veiga, Susana Lima, Luís Silva, Vítor Hugo Carneiro, Mário Pinhão, Arminda Manuela Gonçalves, Maria Teresa Malheiro, Álvaro Miguel Sampaio, José Meireles, António J. Pontes, José Machado

**Affiliations:** 1 MEtRICs Research Center, Campus of Azurém, University of Minho, 4800-058 Guimarães, Portugal; b12058@alunos.uminho.pt (J.V.); susanamrlima@gmail.com (S.L.); a74217@alunos.uminho.pt (L.S.); d6705@dem.uminho.pt (V.H.C.); meireles@dem.uminho.pt (J.M.); 2 Bosch Car Multimedia, Rua Max Grundig, 4705-820 Braga, Portugal; mario.pinhao@pt.bosch.com; 3 DMAT-Department of Mathematics, Campus of Azurém, University of Minho, 4800-058 Guimarães, Portugal; mneves@math.uminho.pt (A.M.G.); mtm@math.uminho.pt (M.T.M.); 4 CMAT-Center of Mathematics, Campus of Azurém, University of Minho, 4800-058 Guimarães, Portugal; 5 IPC—Institute for Polymers and Composites, Department of Polymer Engineering, University of Minho, Campus of Azurém, 4800-058 Guimarães, Portugal; amsampaio@dep.uminho.pt (Á.M.S.); pontes@dep.uminho.pt (A.J.P.); 6 DONE Lab—Advanced Manufacturing of Products and Tools, Campus of Azurém, University of Minho, 4800-058 Guimarães, Portugal

**Keywords:** accelerometers, planar motion, surface mount device, deployment optimization

## Abstract

Machine end-effector kinematic analysis is critical to optimizing transporting components where inertial forces are the main loads. While displacements may be measured with relatively high accuracy in transportation equipment motors, the inertial forces in the transported components are seldom optimized. This is especially relevant in electronic component placement systems, where the components have a wide range of configurations (i.e., geometry and mass) and the deployment dimensional/geometric tolerances are remarkably good. The optimization of these systems requires the monitoring of the real position of the accelerometers relative to the measurement point of interest with sufficient accuracy that allows the assembly position to be predicted instantaneously. This study shows a novel method to calibrate this equipment using triaxial accelerometers on a surface mount machine to measure the end-effector accelerations and velocities in its planar motion. The dynamic equations of the system and the method for integration are presented to address the uncertainty on the exact position of the accelerometer sensors relative to the measuring point of interest exist and allow the position correction to optimize response and accuracy.

## 1. Introduction

The measurement of machine kinematics is a key aspect of understanding its behavior and determining the inertial forces of transported components. In deployment systems, a machine end-effector can be simplified as a rigid body moving through space. Indeed, rigid body tracking has been approached in numerous routes. Inertial Measurement Units (IMU), consisting of a combination of linear accelerometers and gyroscopes, have been used to sense linear accelerations and angular velocities in vehicles, aircraft and satellites [1], with and without distributed control [2]. Martin et al. [3] measured all six degrees of freedom (DOF) accelerations and the kinematics of a rigid body with two methods—one using a set of nine linear accelerometers and three angular rate sensors and another using only three linear accelerometers and three angular rate sensors.

Recent trends show the substitution of angular rate sensors (e.g., gyroscopes) with linear accelerometers, creating an accelerometer’s exclusive configuration [4]. Low-cost IMUs are often limited by gyroscopes defects, such as large size, high cost, large bias instability and drift issues [5].

To measure all six-axis accelerations, at least six linear accelerometers should be used [6,7]. However, the instant angular velocity to measure angular acceleration and using only six accelerometers can lead to the accumulation of errors [6]. To address this issue, six accelerometers can be placed in a precise configuration to mitigate this effect [7], or nine accelerometers can be used to estimate the angular velocities.

Other configurations may also be found, consisting of six [8], eight [9], nine [1,10,11] and twelve [12] linear accelerometers in a single measurement unit. Accelerometers with a single inertial mass were also developed to measure all six-axis accelerations. Ho and Lin [12] designed an accelerometer with only one inertial unit based on the deformation of the dual annular membrane structure. Meng et al. [13] developed an accelerometer based on the parallel mechanism comprising only one proof mass and twelve piezoelectric sensors and Wang et al. [4] presented a comprehensive study of the forward and inverse dynamic equations of this accelerometer.

A higher number of accelerometers can be used to improve accuracy by redundancy. You et al. [14] studied the accuracy and redundancy of large arrays of accelerometers. They show that planar motion (i.e., 3 DOF) is comprised of two translational accelerations and one rotational acceleration. Since it is a subset of the general motion, all of the techniques mentioned above can be applied.

Compared with the general spatial case, the planar motion of a rigid body seems to have less impact in the literature. Madwick et al. [15] studied the geometric requirements and configurations of linear accelerometers to study the general planar motion of a rigid body. They show configurations of two to four accelerometers and present a minimum of four linear accelerometers to study the rotational motion parameters and the general motion (rotation and translation). Williams and Fyfe [16] used seven linear accelerometers to track a planar motion of a dummy head. Five of the seven accelerometers are in-line, and the redundancy is used to determine the angular acceleration, which provides an indication of the data variability and consistency.

In real applications, monitoring the positions of the accelerometer sensors with accuracy is central to avoiding measurement errors. Shea and Viano [17] presented a method for determining the accelerometer sensor position intra-device. This work presents the application of four linear accelerometers to measure linear and angular accelerations/velocities on an industrial machine with planar motion (translation and rotation). The method used to characterize the kinematic behavior is shown to improve the results’ accuracy by optimizing the position of the accelerometer sensors. This optimization is motivated by uncertainties in the exact accelerometer sensor positions after assembly.

This method can be useful when uncertainties about the application parameters exist, and specific results are known. In this study, the end velocities are the known results used to adjust the accelerometer sensor positions in situ to obtain results with an optimized accuracy in an electronic component placement system.

## 2. Materials and Methods

### 2.1. Case Study—Electronic Component Placement System

A Panasonic model NPM-W2 with two gantries, each one with a head of three nozzles, and each was used as an in situ case study. This is an automated, high-speed, high-precision, very complex placement machine. The main operational task of this machine is to pick up electrical components from a feeder, then transport and mount them on printed circuit boards (PCBs) in the surface-mount technology (SMT) production process.

The pickup and transportation of the electric components are executed by nozzles actuated by vacuum. A full cycle of the production process consists of the movement of a machine head: (i) pickup components from an initial feeder position (i.e., a number of components equal to the number of nozzles on the head); (ii) the machine head moves to a camera to check if the electrical components are in good conditions to be mounted; (iii) finally, if the components are well supported, the machine head moves to the corresponding mounting position of each electrical component over the PCB. If there are component support problems, the system rejects those components. The process continues with a new cycle, with the machine head moving to the feeder again to pick up more electronic components.

The correlation between accelerations and the mass properties of the electronic components is the main cause of loads between the nozzle and electronic components. To select and design nozzles capable of picking up, transporting, and mounting the electronic components correctly, the study of those forces and nozzle response is crucial.

The machine head accelerations and velocities are independent of the nozzles and are controlled by two parameters: the Mounting Speed (MS), which controls both the machine translational and angular accelerations, and the Teta Speed (TS), which controls the angular acceleration and velocity of the machine. Other parameters exist but are not relevant to this study.

### 2.2. Experiment

The experiment’s objective was to estimate the nozzles’ accelerations and velocities when transporting an electrical component for the different values of MS and TS.

**Figure 1:**
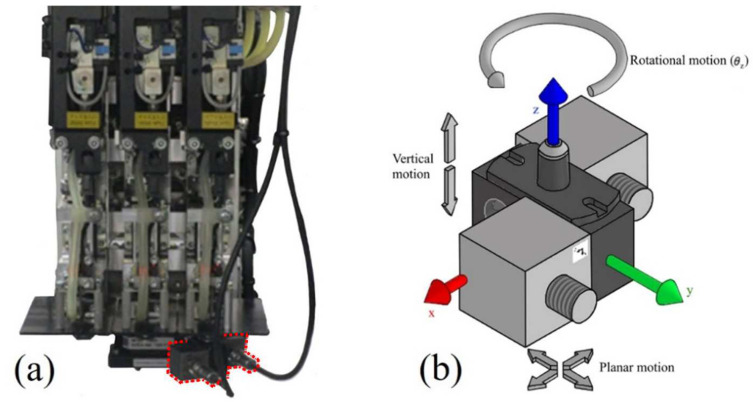
Assembly of nozzle and accelerometers: (**a**) detail of machine head with accelerometers highlighted with a red dotted line. (**b**) Graphical representation of machine/inertial referential frame and possible nozzle movements. Note: The nozzle has translational motion in the *x*, *y,* and *z*-directions and rotational motion in the *z*-direction.

To achieve this, a custom nozzle was built to be mounted on a machine head carrying two triaxial accelerometers. The machine is then set to execute cycles in order to mount a specific component at determined MS and TS. If the assembled nozzle is not able to pick a specific component, when the machine reaches the camera, it detects that it carries no component and moves back to its initial position. Then it executes the cycle two more times, trying to pick up and mount the next component of the same type. Figure 1a shows the assembly of the nozzle with the accelerometers on the machine head.

The tests were executed in situ in an industrial environment with nearby machines working; therefore, the reading data by the accelerometers were expected to display some noise. The machine’s transport system, consisting of gantries, can produce planar motion (both translational and rotational motion) and the machine head can produce vertical translations comprising 4 DOF in total for the motion of the nozzle. Figure 1b shows the assembly of the nozzle and the two accelerometers, the inertial reference frame, and the possible motion of the nozzle. The inertial reference frame directions are based on the machine directions.

The angle $\theta_{z}$ is the nozzle and the inertial frame angle of rotation around the inertial *z*-axis. For simplicity, it was assumed that the nozzle’s first position of every cycle corresponds to (0, 0, 0) and $\theta_{z}$ is equal to zero degrees the nozzle (Figure 1b) relative to the inertial frame, yet that was not the nozzle’s initial orientation.

Two PCB Piezotronics model X3713B1150G triaxial DC capacitive accelerometers were assembled, as shown in Figure 1. The accelerometer’s power and signal condition of their output signals was performed by two three-channel DC signal conditioners, PCB Piezotronics model 478B05. The accelerometers were individually connected to one conditioner, and the conditioners were connected to a Siemens LMS SCADAS mobile hardware. The accelerometer sensors measured the accelerations in three directions and can be represented by their center of mass (CM) since their measurement is relative to their CM position.

**Figure 2:**
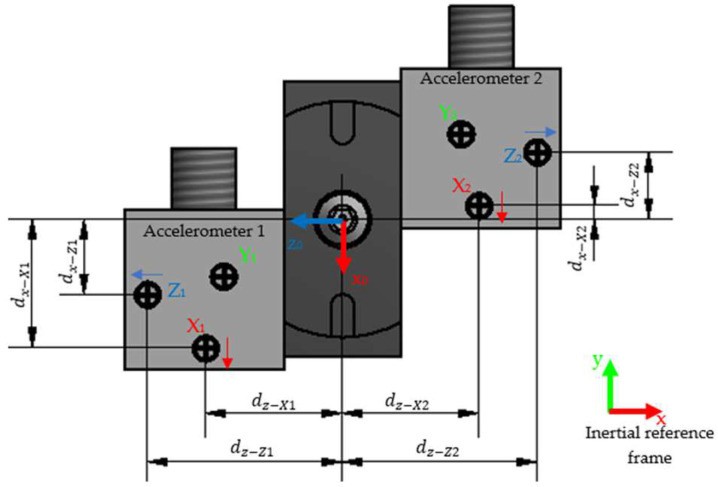
Nozzle and accelerometer assembly: accelerometer positions and their direction of measurement, where nozzle reference framework is (X0,Y0,Z0) and the inertial reference framework is (X,Y,Z). Note: The Y0, Y1, and Y2 directions can be found by the right-hand rule.

The accelerometers’ CM positions are presented in Figure 2, named X1, Y1, Z1 and X2, Y2, Z2. The accelerometer names correspond to their reading direction in their respective accelerometer coordinates, and the number represents to which accelerometer they belong. Their respective positive direction of measurement is represented by an arrow of the same color except for the Y1 and Y2—these directions are vertical and can be easily found by the right-hand rule. This means that if the CM accelerates in the arrow direction, a positive reading is done by the accelerometer sensor.

The nozzle reference frame is also shown in Figure 2 with subscript 0. For $\theta_{z}$ equal to zero degrees, the *x*-nozzle-direction, $x_{0}$, is coincident with the negative *y*-direction of the inertial frame and the *z*-nozzle-direction, $z_{0},$ is coincident with the negative *x*-direction of the inertial frame. This is similar to a 90° rotation of a frame in terms of the *y*-axis of the inertial frame, followed by a 90° rotation of the rotated frame in terms of the *x*-axis of the inertial frame. The nozzle reference frame orientation was chosen to resemble the directions of the accelerometer’s sensors.

It is known that after the cycle, the nozzle position and rotation angle, $\theta_{z}$, are equal to its initial position and orientation. However, the initial orientation was not known before the test. To find it, a motion in only the *x*-axis followed by a motion in only the *y*-axis in inertial reference coordinates was performed after each test. In situ tests were conducted for the parameter MS of 20%, 40%, 60% and 100%, which controls the nozzle accelerations during the production process.

### 2.3. Method

The acceleration of a point on a rigid body is given by Equation (1) [1,6].

(1) $$a_{p}=a_{b}+{\dot{\theta }}_{b}\times \left({\dot{\theta }}_{b}\times p_{p}\right)+{\ddot{\theta }}_{b}\times p_{p}$$

where:

$a_{p}$ = Acceleration of the point relative to the inertial frame.
$a_{b}$ = Acceleration of the body relative to the inertial frame.
$p_{p}$ = Position vector of the point from the origin of the body frame.
${\dot{\theta }}_{b}$ = Angular velocity of the body.
${\ddot{\theta }}_{b}$ = Angular acceleration of the body.

On the application, the nozzle rotations about the ˆ and *y* inertial frame axes ($\theta_{x}$ and $\theta_{y}$, respectively) are fixed; therefore, all its derivatives are zero.

(2) $${\dot{\theta }}_{x}={\dot{\theta }}_{y}={\ddot{\theta }}_{x}={\ddot{\theta }}_{y}=0$$

where ${\dot{\theta }}_{x}$ and ${\dot{\theta }}_{y}$ are the angular velocities and ${\ddot{\theta }}_{x}$ and ${\ddot{\theta }}_{y}$ are the angular accelerations about the inertial frame on the *x*- and *y*-axis, respectively. This application represents a planar motion problem (in the XY inertial plane) plus a vertical translation motion (along the *z*-axis). The planar motion and the vertical motion can be treated independently.

Regarding the planar motion, according to [3,16], the acceleration of a point on a rigid body can be reduced to Equation (3),

(3) $$a_{p}=a_{b}+{\ddot{\theta }}_{z}\cdot s+{\dot{\theta }}_{z}^{2}\cdot p_{p}$$

where s is obtained through a rotation of π/2 radians of $p_{p}$ (about the *z*-axis). An accelerometer sensor reads the acceleration at its center of mass (CM) point in a particular direction (accelerometer sensor axis). If an accelerometer axis direction i is parallel to a unit vector $\sigma_{i}$, the measured acceleration of the accelerometer sensor, $a_{i}$, can be expressed by the product between the accelerations at its CM point and $\sigma_{i}$ as in Equation (3) [3]:

(4) $$a_{i}=\sigma_{i}\cdot a_{b}+{\ddot{\theta }}_{z}\cdot \sigma_{i}\cdot s_{i}+{\dot{\theta }}_{z}^{2}\cdot \sigma_{i}\cdot p_{pi}$$

where $p_{pi}$ is the position of the accelerometer sensor i CM on the body.

For the planar motion, there are 3 DOFs: two translation accelerations ($a_{bx}$, $a_{by}$) and one angular acceleration (${\ddot{\theta }}_{z}$). If only three accelerometer sensors are used, it is possible to see from Equation (3) that solving for the 3 DOFs, for a general case, results in at least one differential equation since equations for $a_{bx}$, $a_{by}$ and ${\ddot{\theta }}_{z}$ are a function of ${\dot{\theta }}_{z}^{2}$. However, it is possible to make a configuration of three accelerometer sensors not dependent on ${\dot{\theta }}_{z}^{2}$. According to Equation (3), such a configuration can be made if the accelerometer sensors axis, $\sigma_{i}$, are perpendicular to their position $p_{pi}$, which geometrically means that each of their axes are tangential to a corresponding circumference of radius $p_{pi}$.

In practical cases, it can be difficult to place the accelerometer sensors with sufficient accuracy. In such conditions, $\sigma_{i}\cdot p_{pi}$ can be considered zero, either due to space limitations, accuracy in placement (misalignments), or restrictions in sensor positioning. The latter comprise those present in this application, caused by using triaxial accelerometers where the internal accelerometer sensors’ relative positions are fixed. In such cases, four accelerometers can be used if the configuration does not belong to an infeasible set as studied by [16], and ${\dot{\theta }}_{z}{}^{2}$ can be treated as a variable creating a system of four equations to four variables. The equations can then be solved for $a_{bx}$, $a_{by}$, ${\ddot{\theta }}_{z}$ and ${\dot{\theta }}_{z}{}^{2}$ allowing an analytical solution that avoids differential equations.

Similar to the general spatial motion as shown in [6], the use of a differential equation for the system model where the angular acceleration is the function of the angular velocity may create unstable results and an accumulation of errors. As small errors may exist in the calculations of $a_{bx}\left(t\right)$, $a_{by}\left(t\right)$ and ${\ddot{\theta }}_{z}\left(t\right)$ at time t, the angular velocity, ${\dot{\theta }}_{z}\left(t\right)$, is also integrated with small errors. On the following time step, $a_{bx}\left(t+\Delta t\right)$, $a_{by}\left(t+\Delta t\right)$ and ${\ddot{\theta }}_{z}\left(t+\Delta t\right)$ depend on ${\dot{\theta }}_{z}\left(t\right)$ and its small error; consequently, ${\dot{\theta }}_{z}\left(t+\Delta t\right)$ is calculated with an accumulation of errors from the previous time-steps. These can build into significant errors over time and can lead to unstable and wrong results.

For this application, the problem is divided into planar motion and vertical translation. The body of the general case is the nozzle in the application. The position vectors of the accelerometer sensors are in the nozzle reference coordinates and are relative to that frame (Figure 2). The nozzle accelerations are also evaluated in nozzle coordinates.

Solving the equations for every CM of the accelerometers is performed by substituting their relative positions by their distance (positive values) to the center of the nozzle (Figure 2), and yields Equations (5)–(10)

(5) $$a_{X1}=a_{x0}+{\ddot{\theta }}_{z}\;\cdot d_{z-X1}-{\dot{\theta }}_{z}{}^{2}\cdot d_{x-X1}$$

(6) $$a_{X2}=a_{x0}-{\ddot{\theta }}_{z}\;\cdot d_{z-X2}+{\dot{\theta }}_{z}{}^{2}\cdot d_{x-X2}$$

(7) $$a_{Z1}=a_{z0}-{\ddot{\theta }}_{z}\;\cdot d_{x-Z1}-{\dot{\theta }}_{z}{}^{2}\cdot d_{z-Z1}$$

(8) $$a_{Z2}=-a_{z0}-{\ddot{\theta }}_{z}\;\cdot d_{x-Z2}-{\dot{\theta }}_{z}{}^{2}\cdot d_{z-Z2}$$

(9) $$a_{Y1}=a_{y0}$$

(10) $$a_{Y2}=-a_{y0}$$

where $a_{X1}$, $a_{X2}$, $a_{Y1}$, $a_{Y2}$, $a_{Z1}$, $a_{Z2}$ are the acceleration reading of the accelerometers X1, X2, Y1, Y2, Z1, Z2, respectively. $a_{x0}$, $a_{y0}$ and $a_{z0}$ are the nozzle accelerations in the x-, y- and z-directions in nozzle coordinates, respectively, ${\dot{\theta }}_{z}$ and ${\ddot{\theta }}_{z}.$ These are, respectively, the angular velocity and angular acceleration of the nozzle.

However, we are interested in measuring the translation/rotational accelerations and velocities of the nozzle in the inertial frame coordinates. A transformation of $a_{x0}$, $a_{z0}$ and $a_{y0}$ to the inertial frame coordinates was also explored.

Regarding the vertical motion, both $a_{Y1}$ and $a_{Y2}$ are used to determine $a_{y0}$ by Equation (11), which is equal to the machine vertical actuation, i.e., the nozzle vertical acceleration in coordinates of the inertial frame, $a_{z-nozzle}$,

(11) $$a_{z-nozzle}=a_{y0}=\left(a_{Y1}-a_{Y2}\right)/2$$

For the planar motion, Equations (5)–(8) are used as a system of four equations to four variables ($a_{x0}$, $a_{y0}$, ${\ddot{\theta }}_{z}$, ${\dot{\theta }}_{z}{}^{2}$) making it possible to solve for the nozzle accelerations and the square of the nozzle angular velocity from Equations (12)–(15).

(12) $$\begin{array}{cc} a_{x0}= & (a_{X1}\cdot \left(d_{z-X2}\cdot \left(d_{z-Z2}+d_{z-Z1}\right)+d_{x-X2}\cdot \left(d_{x-Z2}+d_{x-Z1}\right)\right)\\ & +d_{z-X1}\cdot \left(a_{X2}\cdot \left(d_{z-Z2}+d_{z-Z1}\right)+\left(a_{Z2}+a_{Z1}\right)\cdot d_{x-X2}\right)\\ & +d_{x-X1}\cdot \left(\left(-a_{Z2}-a_{Z1}\right)\cdot d_{z-X2}+a_{X2}\cdot \left(d_{x-Z2}+d_{x-Z1}\right)\right))/\\ & (d_{z-X2}\cdot \left(d_{z-Z2}+d_{z-Z1}\right)+d_{z-X1}\cdot \left(d_{z-Z2}+d_{z-Z1}\right)\\ & +d_{x-X2}\cdot (d_{x-Z2}+d_{x-Z1})+d_{x-X1}\cdot \left(d_{x-Z2}+d_{x-Z1}\right)) \end{array}$$

(13) $$\begin{array}{cc} a_{z0}= & -(a_{X2}\cdot \left(d_{x-Z1}\cdot d_{z-Z2}-d_{x-Z2}\cdot d_{z-Z1}\right)+a_{X1}\cdot \left(d_{x-Z2}\cdot d_{z-Z1}-d_{x-Z1}\cdot d_{z-Z2}\right)\\ & +d_{z-X2}\cdot (a_{Z2}\cdot d_{z-Z1}-a_{Z1}\cdot d_{z-Z2})+d_{z-X1}\cdot \left(a_{Z2}\cdot d_{z-Z1}-a_{Z1}\cdot d_{z-Z2}\right)\\ & +d_{x-X2}\cdot \left(a_{Z2}\cdot d_{x-Z1}-a_{Z1}\cdot d_{x-Z2}\right)+d_{x-X1}\cdot \left(a_{Z2}\cdot d_{x-Z1}-a_{Z1}\cdot d_{x-Z2}\right))/\\ & (d_{z-X2}\cdot \left(d_{z-Z2}+d_{z-Z1}\right)+d_{z-X1}\cdot \left(d_{z-Z2}+d_{z-Z1}\right)\\ & +d_{x-X2}\cdot \left(d_{x-Z2}+d_{x-Z1}\right)+d_{x-X1}\cdot \left(d_{x-Z2}+d_{x-Z1}\right)) \end{array}$$

(14) $$\begin{array}{cc} {\ddot{\theta }}_{z}= & -(a_{X2}\cdot \left(d_{z-Z2}+d_{z-Z1}\right)+a_{X1}\cdot \left(-d_{z-Z2}-d_{z-Z1}\right)+\left(a_{Z2}+a_{Z1}\right)\cdot d_{x-X2}\\ & +\left(a_{Z2}+a_{Z1}\right)\cdot d_{x-X1})/(d_{z-X2}\cdot \left(d_{z-Z2}+d_{z-Z1}\right)+d_{z-X1}\cdot \left(d_{z-Z2}+d_{z-Z1}\right)\\ & +d_{x-X2}\cdot \left(d_{x-Z2}+d_{x-Z1}\right)+d_{x-X1}\cdot \left(d_{x-Z2}+d_{x-Z1}\right)) \end{array}$$

(15) $$\begin{array}{cc} {{\dot{\theta }}_{z}}^{2}= & -(\left(a_{Z2}+a_{Z1}\right)\cdot d_{z-X2}+\left(a_{Z2}+a_{Z1}\right)\cdot d_{z-X1}+a_{X1}\cdot \left(d_{x-Z2}+d_{x-Z1}\right)\\ & +a_{X2}\cdot \left(-d_{x-Z2}-d_{x-Z1}\right))/(d_{z-X2}\cdot \left(d_{z-Z2}+d_{z-Z1}\right)+d_{z-X1}\cdot \left(d_{z-Z2}+d_{z-Z1}\right)\\ & +d_{x-X2}\cdot \left(d_{x-Z2}+d_{x-Z1}\right)+d_{x-X1}\cdot \left(d_{x-Z2}+d_{x-Z1}\right)) \end{array}$$

For the planar motion kinematic analysis, only Equations (12)–(14) are needed, since the angular velocity, ${\dot{\theta }}_{z}$, can be integrated from the angular acceleration, ${\ddot{\theta }}_{z}$. From Equation (15) it is not possible to determine the direction of rotation. Using these equations, the system does not have differential equations, depending only on geometric parameters (distances d) and the accelerometer sensor readings. The nozzle accelerations are transformed to the inertial frame coordinates by Equation (16).

(16) $$\left\{\begin{array}{c} a_{x-nozzle}\\ a_{y-nozzle} \end{array}\right\}=\left[\begin{array}{cc} \cos\left(\theta_{z}+\pi \right) & -\sin\left(\theta_{z}+\pi \right)\\ \sin\left(\theta_{z}+\pi \right) & \cos\left(\theta_{z}+\pi \right) \end{array}\right]\cdot \left\{\begin{array}{c} a_{z0}\\ a_{x0} \end{array}\right\}$$

where $a_{x-nozzle}$ and $a_{y-nozzle}$ are the nozzle accelerations in inertial frame coordinates.

The nozzle velocities ($v_{x}$, $v_{y}$, $v_{z}$, ${\dot{\theta }}_{z}$) in the inertial referential frame are obtained by integrating the translational and angular accelerations using the trapezoid rule. For the position and rotational angle ($p_{x}$, $p_{y}$, $p_{z}$, $\theta_{z}$), the velocities are integrated also using the trapezoid rule. However, a threshold value is used where velocities ($v_{x}$, $v_{y}$, $v_{z}$, ${\dot{\theta }}_{z}$) are less than the threshold in a particular time-step that velocity is assumed to be zero. This avoids position and rotational angle drift during the sections where the nozzle is not moving, but the integrated velocity value is slightly different from zero. This is important since there are significant periods of time when the nozzle was stopped. The model described by Equations (11)–(14) and (16) and the integrations were developed in a Scilab script [18].

The translational acceleration and velocity magnitudes in the XY plane are also estimated by Equations (17) and (18).

(17) $$a=\sqrt{a_{x}^{2}+a_{y}^{2}}$$

(18) $$v=\sqrt{v_{x}^{2}+v_{y}^{2}}$$

The exact position of each sensor inside the accelerometers is not known due to the ± 0.8 mm tolerances and the uncertainty from the exact position of the sensors is significant to the problem. The difference between the distances d used for Equations (12)–(15) and the exact actual distances causes errors in the determination of the nozzle angular and translation accelerations. These errors are accumulated over time in the integrations for nozzle velocities, position and rotational angle.

To enhance the estimation of the nozzle acceleration is necessary to determine the real position of the sensors. For that, a calibration process should be performed for each accelerometer to find the real positions of the sensor’s CM with sufficient accuracy. This method improves the calculated results accuracy by adjusting the distance values of d through known information about the expected response of the application itself. Although no accelerations are known a priori, it is known that at the end of every cycle that the nozzle translational velocities and rotational velocities should be zero since the machine is at rest. The d variables are, therefore, defined as their nominal values (according to the nozzle nominal dimensions and accelerometers nominal sensors CM position—Figure 1) plus a deviation variable e, such as in Equation (19) for the $d_{z-X2}$ distance, where $d_{z-X2}^{nominal}$ is the nominal distance (Figure 2) between the X2 accelerometer sensor and the center of the nozzle. The variable *e* fundamentally represents the deviation error relative to the real instant coordinates.

(19) $$d_{z-X2}=d_{z-X2}^{nominal}+e_{z-X2}$$

This approach adds eight variables ($e_{x-X1}$, $e_{z-X1}$, $e_{x-X2}$, $e_{z-X2}$, $e_{x-Z1}$, $e_{z-Z1}$, $e_{x-Z2}$, $e_{z-Z2}$) to the system of equations. The values of e are estimated by optimizing their values to minimize the error of a cost function. This cost function that calculates the nozzle accelerations from the measured data (Equations (12)–(14) and (16)) integrated into the velocities, position and rotation as described previously. The cost is the sum of the absolute values of the velocities at the end of each cycle since it is known that such values should be zero. The algorithm is described by Equation (20).

Since the nominal distance values of d should be closed to the real distance values, the first guess uses the e values as zeros. To accomplish this, the fminsearch(), a Scilab algorithm [18], was used to minimize the cost function.

(20) $$min_{e_{i}}\;\xi \;\;\;\;\;\;\;s.t.:\;\xi =\left|v_{x}\right|+\left|v_{y}\right|+\left|{\dot{\theta }}_{z}\right|$$

where $v_{x}$, $v_{y}$, ${\dot{\theta }}_{z}$ are obtained by the system equations and the distances d are those from Equation (19).

With this approach, a set of e values are found a priori to better approximate the results to the real behavior. After careful inspections of the results, assuming the optimized e values, the results are accepted if they represent a real expected behavior. For all cycles, this was always the case.

### 2.4. Initial Data Treatment

Before the application of the previous method, it was necessary to do an initial treatment to the data and to estimate the initial orientation, $\theta_{z-initial}$.

For each test, the obtained data were divided into parts, each of them corresponding to one cycle and to each motion in only one direction (five parts in total). An average of the readings on each accelerometer was performed in regions where the nozzle had no acceleration (either known regions of constant velocity or regions where the nozzle is stopped). These were then subtracted from the data of the corresponding accelerometer to remove any bias. This also removes the gravity acceleration reading by the accelerometer sensors Y1 and Y2. Since there are no rotations of the nozzle relative to the *x*- and *y*-axes (Figure 1) of the inertial reference frame, the effect of gravity on the accelerometers’ sensors remained constant during the tests.

Then, the movement data for the single directions were used to estimate the initial orientation, i.e., $\theta_{z-initial}$ angle and inclinations (*x*- and *z*-accelerometer sensors orientation relative to the inertial reference frame’s XY plane). The accelerometer inclinations were found to be small angles and not considered for the analysis. $\theta_{z-initial}$ was found to be between 26.63° and 31.66°; the variation was possible due to the manual mounting process.

## 3. Results and Discussion

**Figure 3:**
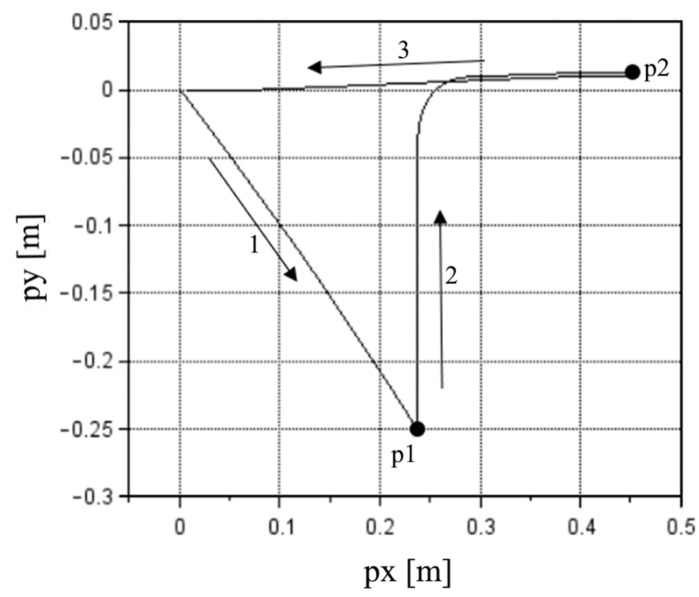
Nozzle route: The first stop is at the feeder at p1, and the second stop (p2) is at the camera. Route based on the results for the first cycle at 100% MS using the optimized e values.

The machine started at the {0, 0} coordinate and moved to the feeder where it stopped, moved to the camera and briefly stopped, then finally returned to its initial position. Figure 3 shows the route of the nozzle using the first cycle at 100% MS as an example, according to the results with the optimized e values where the point of the first stop is p1 (feeder/pickup) and the point of the second stop is p2 (camera).

At p1, the machine executed the pickup of the component; therefore, there was vertical motion while the machine was stopped. However, that motion is not analyzed in this work.

**Figure 4:**
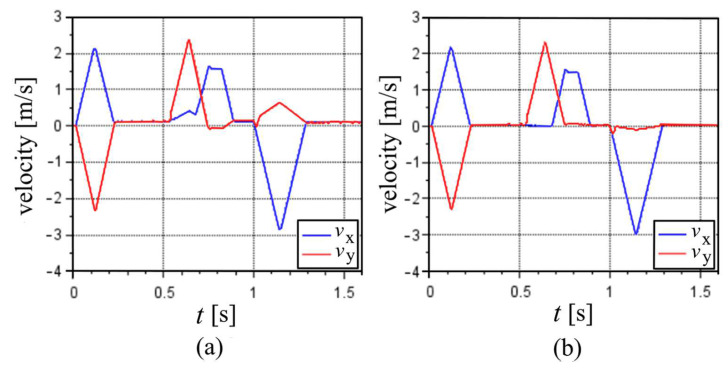
Velocities of the first cycle at 100% MS: (**a**) assuming nominal distances between accelerometer sensors and center of the nozzle, i.e., e values equal to zero. (**b**) Assuming the optimized values for e.

**Figure 5:**
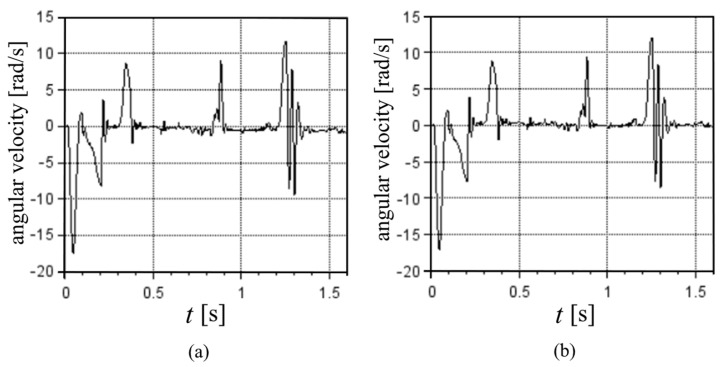
Angular velocity of the first cycle at 100% MS: (**a**) assuming nominal distances between accelerometer sensors and center of the nozzle, i.e., e values equal to zero. (**b**) Assuming the optimized values for e.

To visualize the effect of the corrections of the distances by the optimization of the e values, Figure 4 shows the translational velocity results, and Figure 5 shows the rotational velocity results for the first cycle at 100% MS, where the Figure 4a and Figure 5a consider the nominal distances between accelerometer and the center of the nozzle (e values equal to 0), while Figure 4b and Figure 5b display the optimized e values.

With the optimized values of e, the nozzle velocities were very close to zero in the time periods when the machine was stopped. When e was equal to zero, there were noticeable velocities at those periods, and the results seemed to indicate motions that did not really exist, especially those represented by the *y*-velocity between 1.0 s and 1.3 s (Figure 4a).

**Figure 6:**
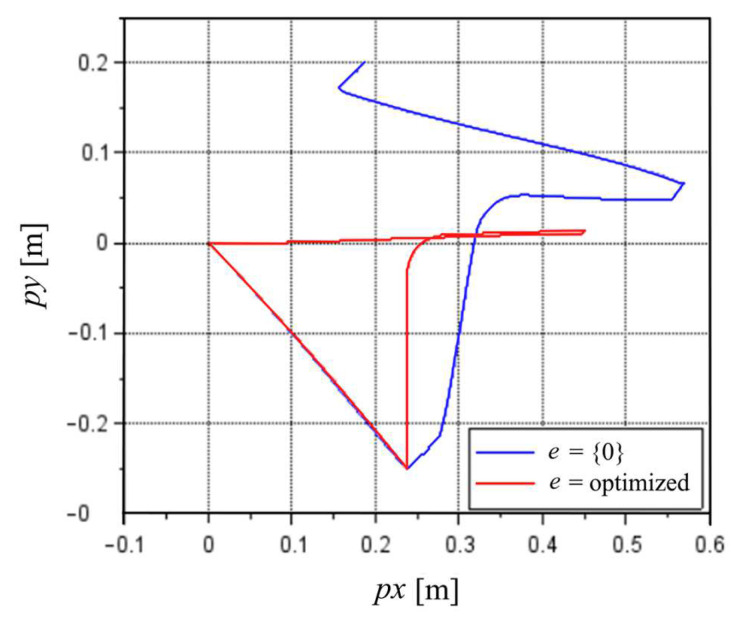
Comparison of the XY plane position results of the nozzle for the first cycle at 100% MS considering e equal to zeros or optimized value of e with the start guess values of zeros.

The impact of the e correction was more noticeable considering the nozzle positions. Figure 6 presents the position of the nozzle in the inertial frame XY plane for the first cycle at 100% MS, considering e equal to zeros or the optimized values with the initial guess of zeros.

With the optimized values of e, the route described by the results resembled the real route with a stop at the feeder of the machine followed by a stop at the camera and back to the initial position. The end position was not equal to {0, 0} as it should have been but was relatively close at {0.008, 0.000} m. Without the e correction, the route was closer to the real route until the stop at the feeder (p1). After that moment, the results were very different from the real route, with very large errors.

**Figure 7:**
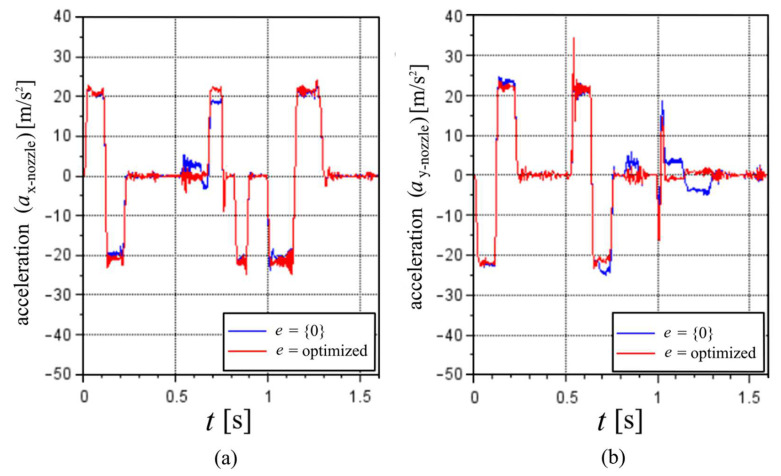
Nozzle acceleration in the inertial frame coordinates, considering e equal to zeros or optimized values with initial guess values of zero in the (**a**) *x*-direction and (**b**) *y*-direction.

Figure 7 shows a comparison between the planar translational accelerations of the nozzle in the inertial frame coordinates. The results are a nine-point moving average to smooth the data, thus reducing the fluctuation and noise. Some differences are noticeable, especially at the maximums and between 0.5 s and 0.7 s for the *x*-acceleration and between 1.0 and 1.3 s for the *y*-acceleration, which leads to the most noticeable difference pointed in the velocities.

**Figure 8:**
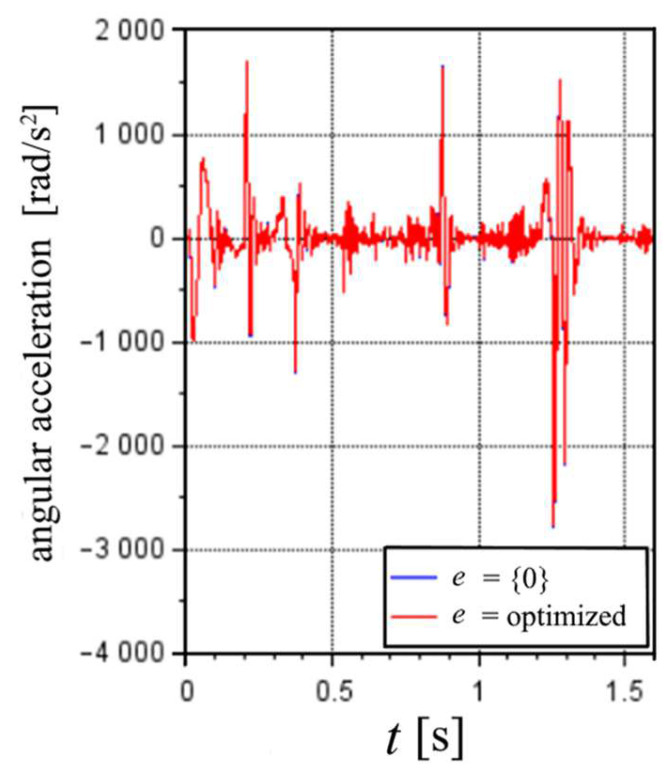
Nozzle angular acceleration, considering e equal to zeros or optimize values with initial guess values of zero.

To conclude the analysis, Figure 8 compares the rotational acceleration of the nozzle. The estimated angular acceleration results suffer from high fluctuations.

Regarding the method, different values were tested for the initial guess of e based on random values between −0.8 mm and +0.8 mm. Different optimized values of e were found, and no relation between the different e optimized values seemed to exist. This means that the e values may not correct the d distances to the values closer to reality, as it was hypothesized. However, even though the optimized e values are different, the results in terms of velocities and route seemed to converge to similar curves.

To show this, Table 1 shows the optimized e values considering the initial guess of zeros and three different sets of initial random values (Rand1, Rand2 and Rand3). The cost of the cost function for the optimized values is also indicated.

**Figure 9:**
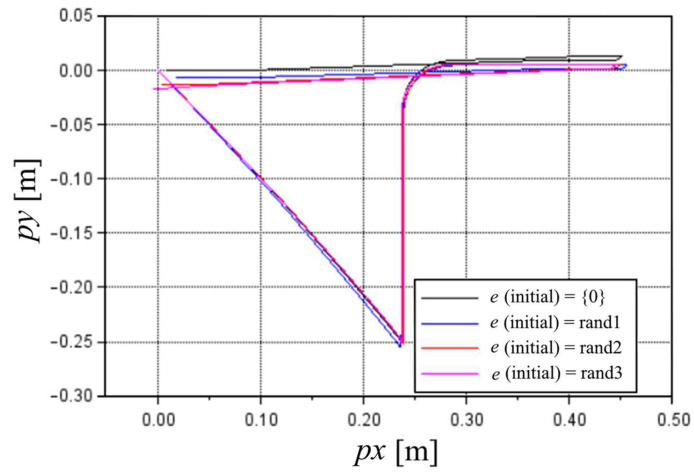
Nozzle route considering different optimized values of e. The first cycle of the 100% MS test.

**Figure 10:**
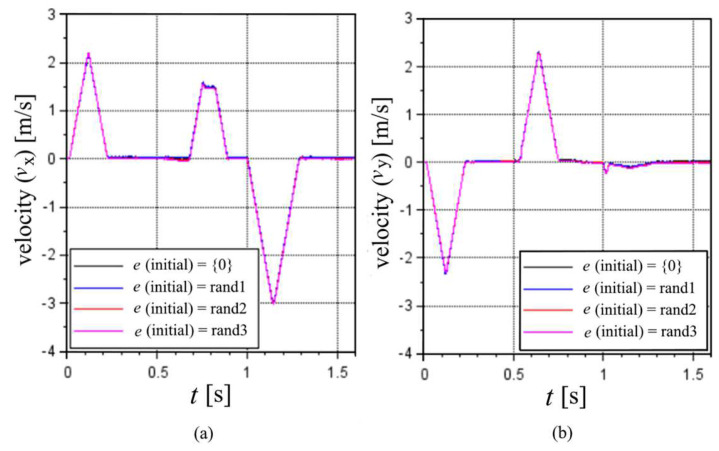
Translational velocities considering different optimized values of e in the (**a**) X axis and (**b**) Y axis, for the first cycle of the 100% MS test.

**Figure 11:**
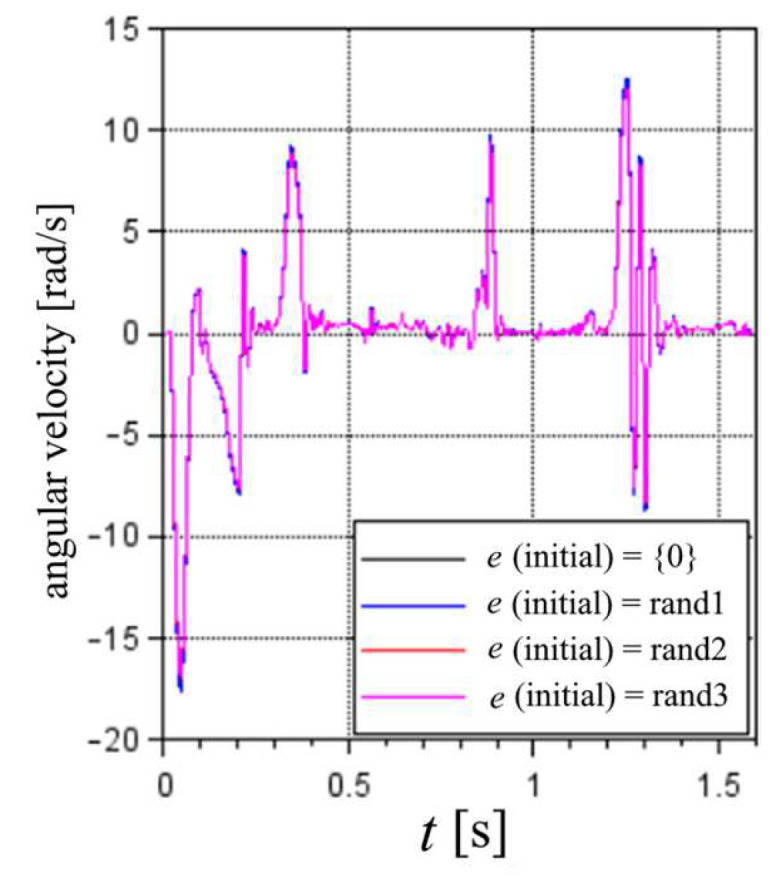
Angular velocity results considering different optimized e values. The first cycle of the 100% MS test.

Figure 9, Figure 10 and Figure 11 show the route, translational velocities, and angular velocity results, respectively, for the optimized e values of Table 1. Figure 9 shows that the optimized routine, even with a random variable start coordinate, can keep to the expected route. The coordinates of the relevant points (p1 and p2) are fully within the initially established ±0.8 mm tolerance.

The results highlighted in Figure 10 show a slight variability; however, all results are a clear and enhanced approximation of the real route, rather than the results not considering a correction of the *d* values by the optimized e values. In terms of velocities, the results present slight differences but are very similar in general. These claims are further supported by the correlations of the translational and angular velocities in Figure 10 and Figure 11.

**Figure 12:**
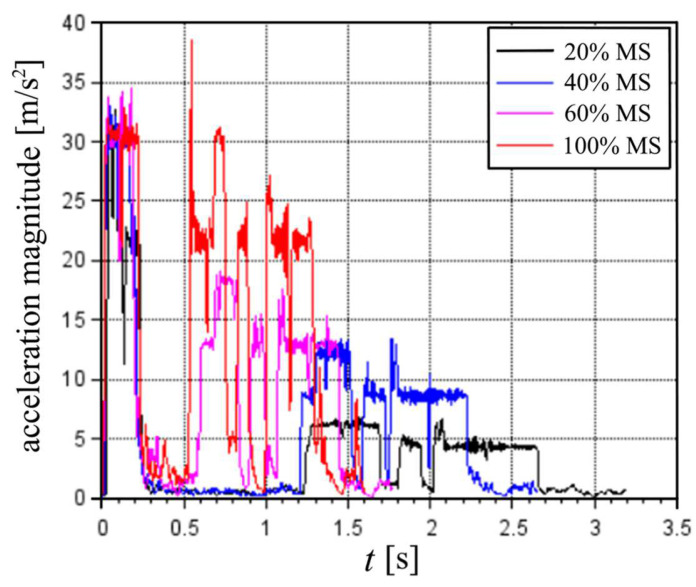
Translational acceleration magnitude for cycles with different MS parameters.

For the rest of the experimental data, the optimized e values were considered for each test using an initial guess of zeros. Regarding the results of the in situ tests, for each cycle, there were clear plateaus of the maximum magnitude of translational accelerations that corresponded to the percentage of the MS parameter relative to the maximum of 100% MS. Figure 12 shows a nine-point moving average with the results of the first cycle of tests at a different MS. For 100% MS, the peak at approximately 0.6 s is not taken into consideration. A similar peak appeared in the vertical acceleration readings and did not seem to result from the machine actuation.

It must be mentioned that each cycle and each test have a slightly different route. This is due to the machine trying to pick up a new component since the previous component was marked as having already been taken. At lower speeds, the machine also executes the vertical pickup motion more slowly, thus increasing the time at rest.

During the first motion, i.e., before 0.5 s, the machine always actuated with maximum acceleration, which is probably due to the fact that no component was being transported. After pickup, i.e., after 0.5 s for 100% and 60% MS and after 1.3 s for 40% and 20%, approximately, the machine supposedly transported a component. Therefore, it was during its second motion that the measurement of the nozzle accelerations was crucial.

The averages of the maximums of acceleration magnitude were estimated for each cycle during its second movement with the same MS parameters, as shown in Table 2.

The maximum estimated acceleration magnitude was 36.00 m/s^2^ during the first movement (before pickup) of a cycle. During the second movement, the highest acceleration estimated was 33.00 m/s^2^ at 100% MS; however, 8 of the 10 cycles at 100% MS showed maximum values between 31 and 32 m/s^2^.

In terms of velocities, there does not seem to be any maximum since the results of the translational velocity magnitude and the angular velocity did not show any plateau. The maximum translational velocity estimated was 3.21 m/s, and the maximum angular velocity estimated was 18.10 rad/s.

## 4. Conclusions

This work presents a method to determine the translational and angular accelerations and velocities of a rigid body undergoing planar motion. This can be used to optimize the results accuracy of the system, i.e., accelerometer positions, based on the known velocities at the end of the studied motions.

The use of accelerometers was shown to be suitable for the stable monitoring of planar translational and angular accelerations of a rigid body. With four accelerometer sensors (i.e., two triaxial accelerometers measuring planar kinematics), the model equations of the system are analytically solvable and not dependent on the angular velocity, therefore avoiding differential equations. While this inverse engineering allows for the estimation of the kinematic parameter, the use of two triaxial accelerometers mitigated the issues related to the experimental apparatus, thus reducing the overall mass, number of components and cables.

The method improves this accuracy and is shown to be promising by approximating the velocities and route curves to the real, expected behavior. Therefore, it is useful for significant uncertainties on the accelerometer’s real positions in highly sensitive applications. The cost function seems to have several local minima depending on the initial guess of e; however, it requires further evaluation to enhance the optimization scheme that can lead to a better estimation of the accelerometer sensors’ positions.

Regarding the results of the application, the maximum translational accelerations could be estimated for each cycle, and approximately corresponded to the percentage of the maximum overall acceleration according to the MS parameter. The tested cycles did not appear to reach a maximum in the velocities. Regarding the angular acceleration, it is proposed that the relationship between the stiffness and inertia of the nozzle plus the accelerometers had a significant impact on the results, making it difficult to estimate the accelerations’ peaks according to the machine actuation.

## Figures and Tables

**Table 1:** Initial and optimized values of e for the first cycle at 100% MS.

*e* Values	Initial e		Rand1		Rand2		Rand3	
	Initial	Optimized	Initial	Optimized	Initial	Optimized	Initial	Optimized
$e_{x-X1}$	0	0.673	−0.620	2.545	0.499	1.656	0.200	0.164
$e_{x-X2}$	0	0.793	−0.532	−0.082	−0.237	0.162	0.327	1.036
$e_{z-X1}$	0	0.478	−0.477	−0.579	0.740	1.558	−0.538	−0.557
$e_{z-X2}$	0	−0.026	0.126	0.074	−0.391	−0.112	0.574	1.652
$e_{x-Z1}$	0	−0.003	−0.547	−0.836	−0.212	−0.213	−0.312	−0.564
$e_{x-Z2}$	0	−0.462	0.301	0.487	−0.648	−0.947	0.361	0.848
$e_{z-Z1}$	0	−1.218	0.046	0.079	−0.319	−0.595	0.730	−2.293
$e_{z-Z2}$	0	−0.441	0.412	0.733	0.309	−0.198	0.251	−0.008
Final cost	0.046		0.058		0.015		0.018	

**Table 2:** Average of maximum acceleration magnitude of every cycle with the same MS parameter.

	Average of Maximum Acceleration Magnitude [m/s^2^]	Real Percentage [%] of 100%
20% MS	6.92	22
40% MS	13.66	43
60% MS	19.17	61
100% MS	31.62	-

## Data Availability

The data presented in this study are available on request from the corresponding author. The data are not publicly available due to being part of an ongoing study.
